# Ependymal cells and neurodegenerative disease: outcomes of compromised ependymal barrier function

**DOI:** 10.1093/braincomms/fcac288

**Published:** 2022-11-04

**Authors:** Diana G Nelles, Lili-Naz Hazrati

**Affiliations:** Department of Laboratory Medicine and Pathobiology, University of Toronto, 1 King’s College Circle, Toronto, ON M5S 1A8, Canada; Department of Paediatric Laboratory Medicine, The Hospital for Sick Children, 555 University Ave, Canada; Department of Laboratory Medicine and Pathobiology, University of Toronto, 1 King’s College Circle, Toronto, ON M5S 1A8, Canada; Department of Paediatric Laboratory Medicine, The Hospital for Sick Children, 555 University Ave, Canada

**Keywords:** ependymal cells, neurodegeneration, brain barrier, cerebrospinal fluid, cilia

## Abstract

Within the central nervous system, ependymal cells form critical components of the blood-cerebrospinal fluid barrier and the cerebrospinal fluid-brain barrier. These barriers provide biochemical, immunological and physical protection against the entry of molecules and foreign substances into the cerebrospinal fluid while also regulating cerebrospinal fluid dynamics, such as the composition, flow and removal of waste from the cerebrospinal fluid. Previous research has demonstrated that several neurodegenerative diseases, such as Alzheimer’s disease and multiple sclerosis, display irregularities in ependymal cell function, morphology, gene expression and metabolism. Despite playing key roles in maintaining overall brain health, ependymal barriers are largely overlooked and understudied in the context of disease, thus limiting the development of novel diagnostic and treatment options. Therefore, this review explores the anatomical properties, functions and structures that define ependymal cells in the healthy brain, as well as the ways in which ependymal cell dysregulation manifests across several neurodegenerative diseases. Specifically, we will address potential mechanisms, causes and consequences of ependymal cell dysfunction and describe how compromising the integrity of ependymal barriers may initiate, contribute to, or drive widespread neurodegeneration in the brain.

## Teaser

Nelles *et al*. report that compromised ependymal barriers in the central nervous system, namely the cerebrospinal fluid-brain barrier and the blood-cerebrospinal fluid barrier, may contribute to the initiation and progression of several neurodegenerative diseases. Evidence suggests that disrupted ependymal barriers dysregulates cerebrospinal fluid homeostasis, alters gene expression, impairs cilia function and modifies ependymal metabolic functions.

## Graphical Abstract

**Graphical abstract:**
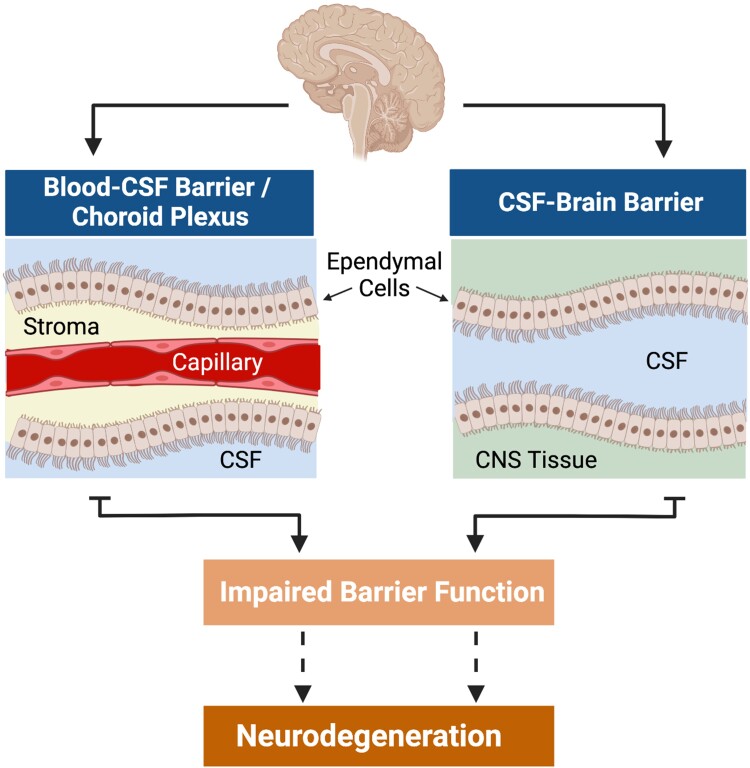

## Introduction

**Figure 1:**
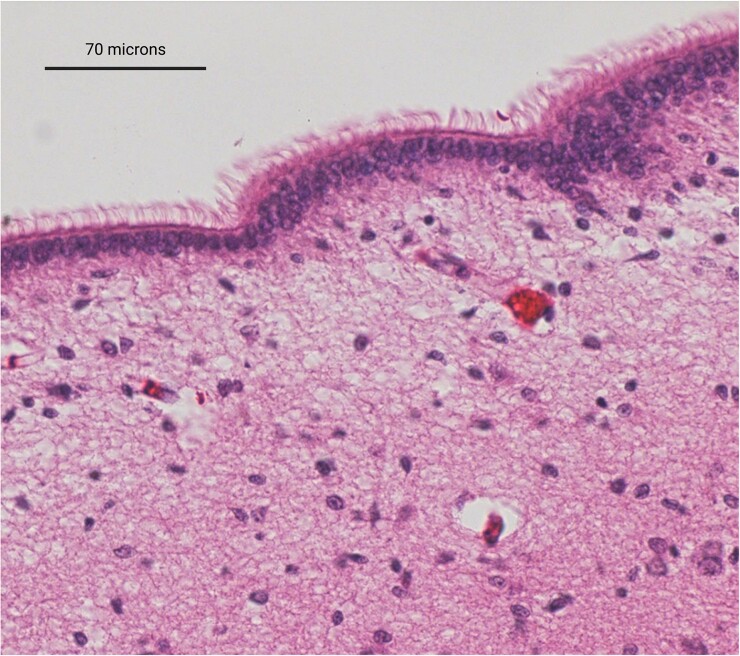
**Photomicrograph of the ependymal cell lining stained with haematoxylin and eosin.** The standard morphological features of ependymal cells include a large oval nucleus, short microvilli, and long cilia that project outwards into the ventricular space. Scale bar represents 70 µm.

Ependymal cells are derived from radial glial cells and consist of a ciliated simple cuboidal epithelium,^1,2^ as shown in Fig. 1. Within the CNS, the polarized ependymal cells form a continuous monolayer that lines the inner surfaces of the cerebral ventricles and the central canal of the spinal cord.^2,3^ This layer creates a cellular barrier between the cavities filled with CSF and the parenchyma of the brain or spinal cord.^4^ Three specific subtypes of ependymal cells have been described based on their structural features and distribution within the CNS.^5,6^ E1 ependymal cells are the most common subtype and are characterized by the presence of multiple motile cilia on their apical surface.^5^ E1 ependymal cells line the bulk of the lateral, third and fourth ventricles, which are located within the forebrain, midbrain and hindbrain, respectively.^5^ E2 ependymal cells are rather biciliated and consist of one primary cilium and one motile cilium, lining select regions of the third and fourth ventricles as well as the spinal canal.^6,7^ E3 ependymal cells are uniciliated and consist of a single primary cilium, mainly occupying a small region of the third ventricle within the recesses of the preoptic area and infundibulum.^5,6^ Ependymal cells are heterogeneous with respect to their cellular dimensions, such that the apical cell membrane can range from 6 × 5 µm to 11 × 8 µm, as observed within the third ventricle,^8^ and the cilia can project up to 20 µm into the ventricular cavities.^9^ Although less well-defined, the spinal ependymal layer that lines the central canal of the spinal cord consists of three main cell types: E2 ependymal cells, tanycytes and CSF-contacting neurons.^10^ Tanycyctes are uniciliated cells with long cellular processes that extend towards the vasculature of the spinal cord parenchyma, whereas CSF-contacting neurons possess mechanosensory properties that resemble retinal bipolar neurons.^10^ Specialized populations of ependymal cells also contribute to the formation of the choroid plexus and line the circumventricular organs.^11,12^

The localization and function of ependymal cells within the CNS ventricular system makes them important cellular barriers that regulate the transport and exchange of molecules between the brain and the body.^13^ Among the three CNS cellular barriers that exist, the most studied barrier is the blood–brain barrier, which is characterized by tight junctions between capillary endothelial cells that control the movement of molecules into the brain to confer protection against foreign substances, blood-borne pathogens and toxins.^14^ Disruption to the blood–brain barrier has been identified as a key component in the pathogenesis of several neurodegenerative diseases and neurological conditions, such as Alzheimer’s disease.^15,16^ The remaining two cellular barriers, namely the blood-CSF barrier and the CSF-brain barrier, are derived from ependymal cells and primarily function to regulate CSF homeostasis and dynamics.^17^ Although some studies have proposed a role of altered ventricular system function and CSF homeostasis in neurodegenerative disease,^18^ ependymal barriers are relatively understudied in the context of disease. In the same way that disruption to the blood–brain barrier may cause neurodegeneration, disruption to ependymal barriers may also contribute to aspects of neurodegeneration.^19^ In this review, we examine potential mechanisms of ependymal cell dysfunction, causes of impaired barrier function and the relationship between ependymal cell dysregulation and the pathogenesis of neurodegenerative diseases. Consequently, these findings will help to establish the role of ependymal cells in neurodegeneration and may encourage the development of targeted treatments and novel diagnostic methods that utilize important biomarkers of ependymal cell dysfunction.

## Ependymal cell functions

### Ependymal barriers

Ependymal cells form the CSF-brain barrier, which is a cellular barrier mediating the bidirectional flow of substances at the interface between the CSF and cerebral or spinal tissue.^20^ The polarized structure and organization of the ependymal cell lining enables the uptake, exchange and removal of biomolecules, signalling factors, ions, metabolites and immune cells between the parenchymal interstitial fluid and the CSF.^2,20,21^ Despite only being a partial barrier due to the lack of tight junctions between individual ependymal cells, the CSF-brain barrier maintains and regulates the composition of the CSF.^22^ Ependymal cells also form components of the blood-CSF barrier, where specialized subtypes of ciliated ependymal cells make up the epithelial layer of the choroid plexus and form an interface between the CSF-filled ventricles and the blood.^23^ Since the endothelium of the choroid plexus is fenestrated, the ependymal cells are linked together by tight junctions in order to precisely control the influx and efflux of molecules between the CSF and circulating blood.^12^ As a complete barrier, the ependymal cells of the blood-CSF barrier provide physical, biochemical and immunological protection to the brain in order to maintain the integrity of CSF dynamics.^24^ Disruption to the blood-CSF barrier may compromise the flow and composition of the CSF, as has been observed in several neuropathologies, such as Alzheimer’s disease and hydrocephalus.^25^ The proper function of both the CSF-brain barrier and the blood-CSF barrier is essential in preserving homeostasis in the brain,^25^ such that damage or disruption to the ependymal cells that form these barriers may initiate, contribute to, or drive neurodegenerative disease.

### Cerebrospinal fluid dynamics

The CSF is produced from specialized populations of ependymal cells within the choroid plexus that form the blood-CSF barrier.^26^ There are three main factors that contribute to CSF equilibrium: the amount of CSF that is produced, that is in circulation, and that can be absorbed at any given time.^27^ The CSF is composed of nutrients, biomolecules, ions, neurotransmitters and endocrine factors, where harmful metabolites, toxins and waste are continually removed from the CSF.^27^ The multiciliated E1 ependymal cells play an important role in regulating the unidirectional flow of CSF via a synchronized cilia beating pattern found on the apical surface facing the ventricular cavities.^28^ The beating pattern of cilia also assists in the distribution of neuroactive hormones and neurotransmitters throughout the CNS.^29^ Not only does ependymal cilium prevent the pooling and accumulation of CSF within the ventricular system, but it provides a large surface area where the CSF can sufficiently interact with the apical surface of the ependymal cell lining in order to facilitate the transport of molecules at the CSF-brain interface.^30^ The steady and continuous flow of CSF enables nutrients, essential biomolecules and signalling factors to be delivered throughout the CNS and for ion homeostasis to be maintained.^30,31^ In addition, a constant flow of CSF facilitates the turnover, detoxification and removal of metabolic waste and toxins that are normally produced by the brain, such as amyloid-beta monomers.^31^ The glymphatic system further contributes to waste removal in the brain, such that metabolic waste and excess fluid is actively removed from the interstitial fluid.^32,33^ The glymphatic system also propels the flow of CSF within the periarterial spaces, thus enabling the contents of the CSF and parenchymal interstitial fluid to be continually exchanged and for waste to be eliminated prior to perivenous drainage.^32,33^ Since the glymphatic system and the ependymal-mediated CSF circuit are closely intertwined,^33^ ependymal cells may play a multifaceted role in cleansing the CSF, though this has not yet been examined. Consequently, the dysfunction of ependymal cilia may impair CSF flow and alter CSF composition, both of which have been linked to several neurodegenerative diseases, such as Alzheimer’s disease and Huntington’s disease.^34^ Since ependymal cells play a major role in maintaining CSF homeostasis, it is likely that ependymal cell dysfunction may underlie aspects of neurodegeneration.

## Ependymal cell neurodevelopment

### Development and maturation

Radial glial stem cells, which are derived from the neuroepithelium, give rise to several types of glial cells and neurons, including ependymal cells and a population of neural stem cells (NSCs), both of which form the neurogenic niche within the ventricular-subventricular zone (V-SVZ).^35,36^ Ependymal cells are typically formed between embryonic Day 16 and 18, with the ependymal cell lining fully complete between 26 and 28 weeks gestation and fully differentiated by the first postnatal week.^1,2,4^ The immature embryonic ependymal cells synthesize and secrete a plethora of neurodevelopmental factors and morphogens to support neurogenesis, differentiation, axonal pathfinding and guidance, and NSC self-renewal.^37,38^ Specifically, these secretions include epidermal growth factor, fibroblast growth factor 2, noggin, Notch-1, bone morphogenic protein, ephrins and pigment epithelium-derived factor.^39–41^ During development, ependymal cells and NSCs reorganize themselves along the ventricular walls into pinwheel structures called rosettes, such that the ependymal cells form an outer shell around the NSCs.^1,6^ During maturation, ependymal cells develop defining structures, such as cilia and adhesion molecules, which enables ependymal cells to function as independent units as well as collectively with neighbouring ependymal cells to form the ependymal cell barrier.^1,6^ The cilia of embryonic ependymal cells are particularly important in propelling embryonic CSF throughout the developing ventricular system and generating a morphogen gradient that drives neuroblast migration.^37^ Expression of the genes *FOXJ1* and *SIX3* is essential in promoting ependymal ciliogenesis and postnatal maturation.^42,43^

### Stem-cell-like properties

In the adult, a subset of ependymal cells is found within the V-SVZ, which is a pluripotent stem cell niche within the lateral ventricles that is responsible for neurogenesis and self-renewal of NSCs in the fully developed brain.^38^ Other components of the V-SVZ include neuroblasts, astrocyte-like NSCs and blood vessels.^44^ Although ependymal cells are associated with the stem cell niche, it remains unclear if ependymal cells possess stem cell-like properties.^45^ While some evidence supports the idea that ependymal cells behave as NSCs,^46^ other studies suggest that ependymal cells lose their stem cell-like features upon maturation^1^ and are not involved in adult neurogenesis.^47^ Recent single-cell RNA-sequencing data further supports the claim that ependymal cells do not possess the same stem cell-like properties that are unique to NSCs.^48^ However, it is important to note that ependymal cells regulate NSC activity through paracrine signalling pathways, such as through the expression of noggin.^40^ The same mechanism that enables ependymal cilia to direct CSF flow likely also drives neurogenesis within the adult,^49^ where the synchronized cilia beating pattern promotes neuroblast migration towards the olfactory bulb.^37^ In contrast to the V-SVZ, the spinal ependymal layer within the central canal has been identified as an endogenous NSC layer due to its regenerative ability in response to mechanical injury of the spinal cord.^10,50^ Consequently, the role of ependymal cells in adult neurogenesis is vague and requires further research in order to determine if ependymal cell recovery or replenishment can be achieved in the brain following injury or dysfunction.

## Structural features of ependymal cells

### Ependymal cilia

Cilia are hair-like structures located on the apical surface of ependymal cells that function in several physiological and developmental processes.^51^ Cilia can be further classified based on their structure and function as either primary or motile cilia.^51,52^ Primary cilia are immotile projections from the cell surface and function in sensory perception of the extracellular environment and signal transduction.^52^ Rather, motile cilia coordinate a synchronized beating pattern in order to regulate CSF circulation, consisting of a complex cytoskeleton made up of axonemes and a ciliary membrane.^27^ Primary cilia is typically organized in the ‘9 + 0’ axoneme configuration, whereas motile cilia are generally found in the ‘9 + 2’ axoneme configuration.^9^ Thus, cilia morphology and function are used to categorize ependymal cells into different subtypes. As previously described, E1 ependymal cells contain multiple motile cilia, E2 ependymal cells contain one motile cilium and one primary cilium, and E3 ependymal cells contain one primary cilium.^5,6^ Since the motile cilia of E1 ependymal cells regulates CSF flow, the dysfunction of E1 ependymal cells would likely impair the unidirectional flow of CSF within the ventricular system. With the cilia of E2 and E3 ependymal cells primarily functioning to sense the CSF composition and transmit extracellular signals, dysfunction of these subtypes would likely alter CSF homeostasis and hinder signalling pathways. Consequently, damage to ependymal cilia or mutations in the genes that control cilia functionality would likely impair the production, circulation and reabsorption of CSF.^20^ However, it remains unclear as to how each specific subtype of ependymal cell contributes to the development of neurodegenerative disease and if additional factors aside from compromised cilia might also impair CSF dynamics and homeostasis to the same extent.

### Ependymal channel proteins

The inherent polarity of ependymal cells drives the specific localization of protein channels and transporters that function to regulate ependymal metabolic processes and maintain CSF composition.^2^ Several glucose transporters (GLUT), such as GLUT1, GLUT2, GLUT3 and GLUT4 are expressed on the apical surface of ependymal cells and facilitate the transport of glucose across the CSF-brain interface.^53–55^ The urate transporter 1 is also localized to the apical surface and is involved in the transport of uric acid between the interstitial fluid and the CSF.^56^ It has been proposed that uric acid contributes to the synchronized beating pattern of cilia and helps to maintain the flow of CSF.^56^ In general, ependymal cells express a variety of anion and cation transporters and channels, such as the sodium-glucose cotransporter 2 and the Na ^+^ -K^+^ ATPase (sodium-potassium ATPase pump), which are essential in controlling CSF ion homeostasis.^57,58^ Additionally, ependymal cells express connexin proteins which aggregate to form gap junctions, functioning to couple electrical and metabolic signals among individual ependymal cells and to coordinate a synchronized cilia beating pattern required for the bulk flow of CSF.^22,59^ Aquaporin channels are highly concentrated on the basolateral aspect of ependymal cells, reaffirming the critical role of water influx and efflux to maintain an osmotic gradient.^60^ Specifically, aquaporin 4 mediates the flow of water between cerebral interstitial fluid and the CSF, such that its dysregulation has been implicated in edema and hydrocephalus.^61^ Altogether, these protein transporters and channels work together to regulate metabolic processes and sustain the composition of CSF.

### Ependymal anchoring structures

The integrity of the ependymal cell monolayer that forms the CSF-brain barrier is maintained by adherens junctions.^62^ At the apical-lateral interface between adjacent ependymal cells, prominent adherens junctions are formed by extracellular homotypic cadherin interactions and intracellular interactions between cadherins and catenins.^63^ The F-actin cytoskeleton provides an anchoring point for adherens junctions and enables the distribution of mechanical and tensile forces so that the ependymal cells can withstand the forces generated from the beating pattern of the cilia.^20,64^ Additionally, the F-actin network is involved in coordinating cilia movement at the apical surface.^64^ At the CSF-brain interface, the ependymal cell layer forms a partial barrier due to the presence of incomplete tight junctions which enables paracellular diffusion of solutes.^65^ However, specialized ependymal cells of the choroid plexus that form the blood-CSF barrier do contain tight junctions.^65^ Disruption to the tight junctions at this interface likely impairs molecular homeostasis and increases the permeability of this barrier for larger biomolecules and immune cells, as is seen in several neurodegenerative diseases.^20^

### Ependymal secretions

Specialized ependymal cells that comprise the choroid plexus and line the circumventricular organs possess high secretory abilities.^66^ Although the ependymal lining of the choroid plexus secretes the majority of the CSF, the ependymal lining of the circumventricular organs, such as the subcommissural organ (SCO), secretes additional factors involved in CSF homeostasis.^66^ These ependymal cells secrete the glycoprotein SCO-spondin into the CSF, such that the aggregation of SCO-spondin molecules form a Reissner’s Fiber.^67^ While Reissner’s Fiber and SCO-spondin play an important early developmental role in commissural axonal pathfinding and guidance,^68^ Reissner’s Fiber is speculated to be involved in osmoregulation and detoxification. This is likely a result of its ability to bind to and remove neurotoxins and neurotransmitters from the CSF while strongly propelling the CSF through the cerebral aqueducts.^69^ Since SCO-ependymal cells that lack the ability to secrete SCO-spondin have been associated with hydrocephalus, the secretory role of these ependymal cells remains essential in maintaining CSF homeostasis.^69^

## Ependymal cell dysfunction in neurodegenerative disease

### Multiple sclerosis

Multiple sclerosis is a neurodegenerative and autoimmune disease characterized by the demyelination of nerve fibres in the CNS, chronic inflammation, cerebral atrophy and axonal loss.^70^ Researchers have recently proposed a link between ependymal cell dysfunction and the pathogenesis of multiple sclerosis.^71^ Indeed, ependymal cells are particularly sensitive to inflammation^71,72^ and develop morphological abnormalities, referred to as the ‘Dot-Dash’ sign, which is believed to be induced by inflammation.^73^ The ‘Dot-Dash’ sign correlates with ependymal perivenular inflammation—an early histopathological event in multiple sclerosis—which is a more sensitive biomarker for the early diagnosis of multiple sclerosis via MRI technology.^73^ Thus, inflammation-induced damage to the ependymal cell lining may reflect a potential origin of the pathogenesis of multiple sclerosis. The altered CSF composition that is observed in multiple sclerosis patients also supports the idea that ependymal cells become dysfunctional.^71,72^ This is likely attributed to the fact that ependymal cells regulate CSF homeostasis and are the only cell type in constant direct contact with the CSF,^71^ such that their dysregulation may alter CSF flow and composition. The CSF of multiple sclerosis patients becomes laden with pro-inflammatory factors, such as the chemokine CXCL12, interleukins IL-22 and IL-2, tumour necrosis factor and IFNγ (interferon-γ),^74^ although it remains unclear if dysfunctional ependymal cells secrete these factors into the CSF or if these factors accumulate in the CSF as a result of impaired waste clearance. While the mechanism by which ependymal cells become dysfunctional is unknown, the dysfunction of ependymal cells may be reflected through upregulation of the cytokine receptors CXCL12 and IFNγ, both of which are involved in inflammatory response cascades.^71^ Similarly, inducible nitric oxide synthase, which is a downstream mediator of inflammation, is upregulated within the ependymal cell lining and has been associated with plaque formation, tissue damage and demyelination.^75^ Additionally, ependymal cell loss or death may be mediated by Fas binding interactions that occur at the ependymal cell surface with infiltrated helper T-cells.^48^

### Alzheimer’s disease

Alzheimer’s disease is a progressive neurodegenerative disease characterized by the deposition of extracellular amyloid plaques, intracellular neurofibrillary tangles, atrophy of neurons and synaptic loss.^76^ One hypothesis of Alzheimer’s disease pathogenesis is the amyloid cascade hypothesis, which states that the accumulation and deposition of amyloid plaques initiates neurodegeneration and drives the downstream formation of neurofibrillary tangles.^77^ Since ependymal cells are essential for the removal of metabolic waste and protein aggregates from the CSF, particularly amyloid-beta monomers, ependymal cell dysfunction may impair the normal CSF detoxification process and cause amyloid neurotoxicity.^78^ Indeed, ependymal cell dysfunction may promote an environment that favours the oligomerization of amyloid-beta monomers into amyloid plaques, which is a molecular event thought to precede and propel the formation of neurofibrillary tangles.^79,80^ Deposits of amyloid plaques and neurofibrillary tangles have been observed within the periventricular regions in Alzheimer’s disease patients, thus reflecting an imbalance between the production and clearance of amyloid plaques at the ependymal cell lining.^81,82^ One receptor type that becomes dysregulated in ependymal cells is the receptor for advanced glycation endproducts, where its upregulation is believed to sustain the influx of glycated amyloid-beta monomers into the brain and promote the formation of amyloid plaques.^83^ In addition, researchers have proposed a link between impaired lipid metabolism and Alzheimer’s disease pathogenesis, where this has been observed within the ependymal cell lining.^84^ Triglyceride lipid droplets rich in oleic acid were found to accumulate within ependymal cells in both a triple-transgenic Alzheimer’s disease mouse model and post-mortem brains of Alzheimer’s disease patients, where subsequent inhibition of NSC regeneration was observed within the mouse model.^85^ Even though lipid droplets are abundantly present in ependymal cells under non-pathological conditions, this amount is significantly increased in Alzheimer’s disease.^86^ The mechanism by which excess lipid droplets accumulate in ependymal cells and the implications of retaining excess lipids with respect to the neuropathology of Alzheimer’s disease in humans is yet to be elucidated.^87^ Similar to multiple sclerosis, widespread neuroinflammation and oxidative stress are also key factors in the pathogenesis of Alzheimer’s disease and may cause ependymal cells to become pathogenic as a result of their sensitivity to inflammation,^88^ though this has been more broadly studied in glial cells.

### Parkinson’s disease

Parkinson’s disease is defined by neurodegeneration of dopaminergic neurons within the substantia nigra and is associated with both motor symptoms (bradykinesia, resting tremor and rigidity) and non-motor symptoms (cognitive decline, neuropsychiatric disorders and insomnia).^89^ Although the role of ependymal cells in the pathogenesis of Parkinson’s disease has not been a focal point in Parkinson’s disease research, some studies suggest a link between ependymal cell dysfunction and Parkinson’s disease.^90^ Deposits of aggregated alpha-synuclein within subependymal tissues and ependymal cells have been observed in Parkinson’s disease patients and correspond with the Braak stages 5 and 6,^90^ which is a well-established staging system of the progression of Parkinson’s disease.^91^ Loss of function mutations in the parkin co-regulated gene (*PACRG*) have also been linked to the pathogenesis of Parkinson’s disease, where *PACRG* is a gene involved in the formation of the axoneme microtubule structure in motile and non-motile cilia.^92,93^ Since *PACRG* is highly expressed and localized to the ependymal cilia lining the ventricular system,^93^ loss of function mutations in *PACRG* may reflect impaired ependymal cilia function, which may contribute to the pathogenesis of Parkinson’s disease. However, it remains unclear if damage to ependymal cells causes dysregulation of *PACRG* or if mutations in *PACRG* driven by genetic or environmental factors are able to trigger ependymal cell dysfunction. Dysregulation of lipid metabolism has also been identified as a driving factor in the deposition of alpha-synuclein and pathogenesis of Parkinson’s disease,^94^ although this has not been specifically examined in ependymal cells.

### Huntington’s disease

Huntington’s disease is a progressive and inheritable neurodegenerative disorder caused by excessive repeats of the cytosine-adenine-guanine codon within the *HTT* gene.^95^ Recently, the role of ependymal ciliary function and ciliogenesis has been identified as a potential pathogenic mechanism of Huntington’s disease.^96^ In both *HTT* knockout mice and patients with Huntington’s disease, ependymal cells displayed delocalized pericentriolar material 1 (PCM1), disorganized cilia, altered CSF flow and hydrocephalus.^97,98^ Indeed, the HTT-PCM1-HAP1 (Huntington-associated protein 1) pathway is thought to regulate ciliogenesis and CSF homeostasis, where mutant mice lacking *HTT* exhibited an uncoordinated cilia beating pattern, abnormal CSF production and circulation, and altered ciliogenesis.^97,98^ These observations suggest a role of the *HTT* gene in regulating ependymal ciliary function,^99^ although it is not fully understood if dysfunctional cilia is an early molecular event in the pathology of Huntington’s disease.

### Traumatic brain injury

Traumatic brain injury (TBI) occurs with an external physical assault to the head that disrupts normal brain functions.^100^ Some studies have found that damage to the ependymal cell lining may contribute to the pathology of TBI.^101^ Ependymal ciliary loss has been observed in TBI mouse models and was found to decrease ventricular CSF flow and alter nutrient-waste exchange.^102^ Mild TBI (mTBI) or concussion may also induce DNA damage in ependymal cells, among other types of glial cells.^103^ In cases of mTBI, expression of γH2AX, a marker of double-stranded DNA breaks, was observed in the ependymal cell lining of the ventricles.^104^ Interestingly, the presence of γH2AX within the ependyma was observed in cases of mTBI displaying clinical symptoms but lacking a neuropathological diagnosis.^104^ These findings suggest that DNA damage in the ependymal cell lining may be an early molecular event post-mTBI and may precede neuropathological diagnosis.^104^ Commonly observed clinical symptoms of TBI include psychiatric and mood disorders, where altered gene expression of p11 in ependymal cells has been recently linked to reduced CSF flow and stress-induced depression.^105^ Thus, ependymal cells may be implicated in both the pathogenesis and clinical presentations of TBI. In addition, TBI has been associated with an increased susceptibility of developing neurodegenerative diseases later in life, particularly Alzheimer’s disease.^106–108^ However, limited research has specifically explored how mTBI-induced DNA damage in the ependymal cell lining may contribute to neurodegeneration.^108^ Nonetheless, these findings suggest that ependymal cells are sensitive to TBI and their dysfunction may initiate the pathogenesis of long-term brain dysfunction associated with TBI.

### Hydrocephalus

Hydrocephalus is a condition characterized by the buildup of CSF within the ventricles leading to ventriculomegaly, where enlarged ventricular spaces hinder the flow of CSF.^109^ Hydrocephalus can be classified as primary (congenital), secondary (acquired externally) or idiopathic, however, the genetic factors underlying primary hydrocephalus suggest a role of ependymal cilia in its pathogenesis.^27^ As previously described, ependymal cilia generate forceful synchronized beats that direct CSF circulation within the ventricular system. Indeed, loss of function mutations in *MDNAH5*, a gene that regulates axonemal dynein, has been linked to primary ciliary dyskinesia and hydrocephalus in mice.^110^ Similarly, mutant mice strains for the *HYDIN* gene, which regulates ciliary motility and fluid transport in the brain, resulted in ciliary immobility and hydrocephalus.^111^ Overall, the biogenesis and maturation of cilia is regulated by *FOXJ1*, *MCIDAS* and *RFX3*, three master transcription regulators that are highly expressed and tightly regulated in ependymal cells.^112^ These studies suggest that functional ependymal cilia is critical for maintaining CSF homeostasis within the ventricular system, such that dysfunction may lead to phenotypes of hydrocephalus.

### Amyotrophic lateral sclerosis

Amyotrophic lateral sclerosis (ALS) is a neurodegenerative motor neuron disease resulting in the loss of motor control.^113^ In a recent single-cell RNA-sequencing study looking at different molecular pathways observed in superoxide dismutase 1 mouse models of ALS, genes involved in the transport of toxins in ependymal cells were differentially expressed.^114^ In cases of sporadic ALS, the CSF of ALS patients contained neurotoxic compounds and inflammatory cytokines, which is thought to be a result of ependymal cell dysfunction.^115^ Isolated and cultured motor and spinal cord neurons displayed signs of ALS-specific neurodegeneration when exposed to the CSF extracted from ALS patients,^116,117^ thus reflecting altered CSF dynamics in ALS patients. Since ependymal cells facilitate toxin removal from the CSF, ependymal cell dysfunction may underlie this phenotype. To further support the implication of ependymal cells in ALS pathogenesis, a novel regimen designed to target and reverse the outcomes of dysfunctional ependymal cells slowed ALS progression in four individuals.^115^ However, the sheer number of studies regarding the role of ependymal cells in ALS progression is limited, and this potential relationship merits further research.

## Ependymal cells in senescence and aging

### Aging

**Figure 2:**
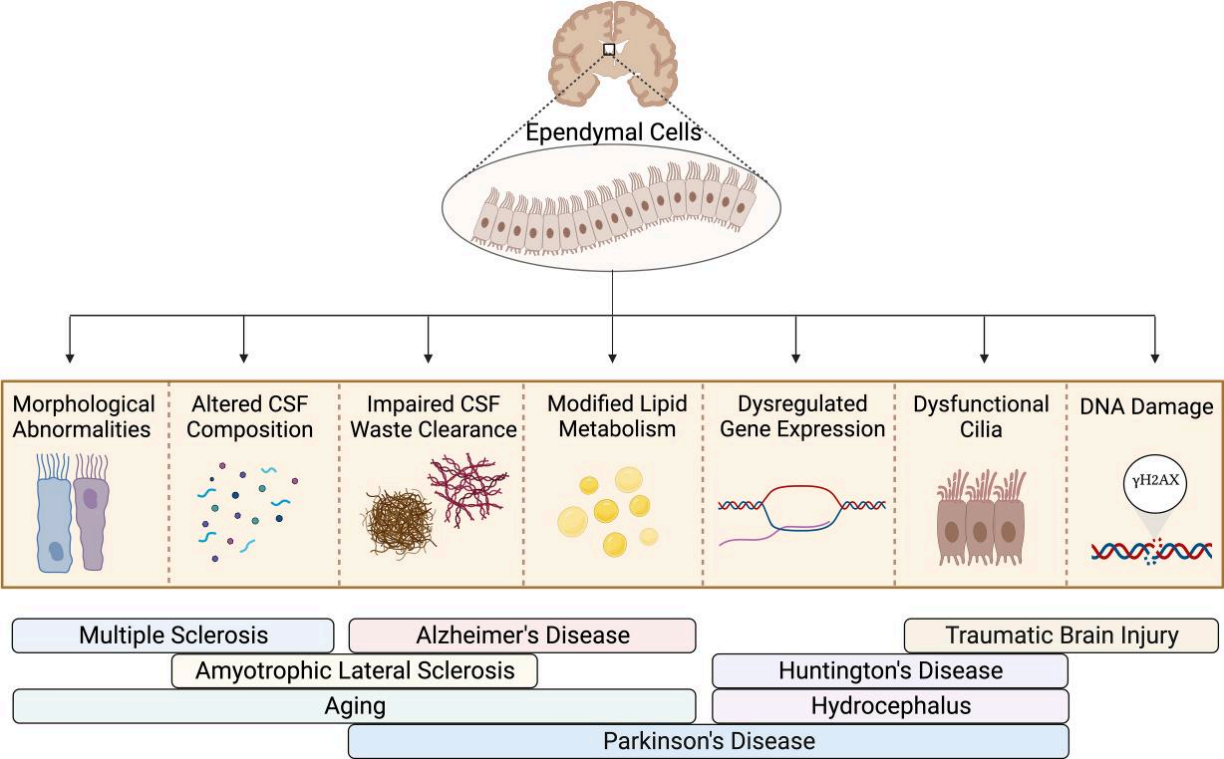
**The manifestation of ependymal cell dysfunction across several neurodegenerative diseases.** Ependymal cell dysfunction presents in several forms across various neurodegenerative diseases, with some diseases sharing common characteristics of ependymal cell dysfunction. In multiple sclerosis, ependymal cell dysfunction presents with morphological abnormalities (the ‘Dot-Dash’ sign) and the infiltration of inflammatory factors in the CSF. Alternatively, ependymal cell dysfunction in Alzheimer’s disease has been linked to impaired clearance of amyloid-beta proteins and neurofibrillary tangles from the CSF and the accumulation of lipid droplets within the cytoplasm. Rather, ependymal cell dysfunction in amyotrophic lateral sclerosis primarily concerns CSF dynamics, displaying both altered CSF composition and impaired CSF detoxification. Interestingly, natural neurodegenerative processes in aging are associated with all four of these dysfunctional ependymal cell phenotypes. Huntington’s disease and hydrocephalus exhibit similar features of ependymal cell dysfunction, both presenting with altered gene expression and compromised cilia function. Although traumatic brain injury is also accompanied by the loss of ependymal cilia, DNA damage (γH2AX) is observed within the ependymal cell lining. In contrast, ependymal cell dysfunction in Parkinson’s disease is multifaceted and has overlapping characteristics with several other neurodegenerative diseases, such that impaired CSF waste clearance is present alongside alterations in gene expression, cilia function and lipid metabolism.

As demonstrated across several neurodegenerative diseases, the dysfunction of ependymal cells is multifaceted and has several molecular presentations, as shown in Fig. 2. However, there are also numerous molecular, metabolic and cellular changes within the ependymal cell lining that are associated with the normal aging process.^118^ Among these changes are the loss of ependymal cells within the V-SVZ, morphological abnormalities, altered protein expression and dysregulated lipid metabolism, which is thought to impair lipid-raft and lipid-trafficking interactions.^119,120^ The progressive loss and thinning of ependymal cells in aging is met with an increase in migratory astrocytes that interpose themselves within the ependymal cell layer and form junctions with the remaining ependymal cells.^121^ In a model of aging mice, ependymal cells accumulated lipid droplets within the cytoplasm, which reflects either metabolic dysregulation or increased uptake of lipids from either the CSF or interstitial fluid of the brain.^119^ Specifically, a reduction in myristoylated alanine-rich C-kinase substrate expression resulted in a decline in ependymal lipid metabolism, accumulation of lipid droplets, oxidative stress, inflammation, and infiltration of reactive microglia, macrophages and astrocytes.^120,122^ In addition, both astrocytes and ependymal cells develop dense bodies, accumulate intermediate filaments and upregulate inflammatory markers.^123^ The morphological abnormalities include tangled or absent ciliary tufts on the apical surface of ependymal cells within several ventricular regions in aging mice,^123^ therefore negatively impacting the flow of CSF. Interestingly, a recent study found that the reduced flow of CSF is linked to the onset of cognitive deficit in healthy elderly individuals,^124^ thus suggesting that natural alterations in CSF homeostasis occur with aging. The reduced production of CSF and the buildup of waste products in the CSF were also found to initiate age-related dementia in adults.^125^ Additionally, neurocognitive decline has been associated with the loss of ependymal cells and periventricular and ventricular abnormalities.^126^ The relationship between CSF dynamics and cognitive deficits indicates the importance of ependymal cell function in preserving overall cognition. Consequently, it is believed that natural dysfunction of ependymal cells occurs during the normal aging process, where this dysfunction may be pre-mature or exacerbated in neurodegenerative diseases.

### Senescence

Cellular senescence is characterized by cell-cycle arrest, the inability to further proliferate and persistent inflammation.^127^ Senescence is a process that occurs naturally with age or it can be triggered with continuous activation of the DNA-damage response pathway in response to unresolved DNA damage.^128^ Senescent cells typically develop a senescence-associated secretory phenotype (SASP), in which cells secrete pro-inflammatory interleukins, chemokines and cytokines into the environment, with prominent markers of senescence being IL-6, IL-1, IL-8, insulin-like growth factor and soluble matrix metalloproteinase.^129^ Senescence has been described as a contributing factor in several neuropathologies.^130^ In the case of mTBI-induced DNA damage within ependymal and other glial cells, the high expression of inflammatory SASP-related factors and morphological markers of senescence suggests that senescence occurs post-mTBI.^104^ Since senescence is believed to initiate a positive feedback loop where the SASP drives a cycle of chronic DNA damage and inflammation,^131^ senescent ependymal cells may secrete SASP factors into the CSF, thus altering CSF composition and flow while potentially spreading senescent signals to nearby brain regions.^104^ Senescence has also been found to alter the CSF composition in age-related dementias,^125^ such that the CSF becomes enriched with tau and ferritin.^132,133^ Since the dysfunction of ependymal cells is known to alter CSF composition and waste removal, ependymal cells that become senescent likely become dysfunctional. In addition, several hallmark phenotypes of multiple sclerosis, such as chronic inflammation and oxidative stress, are well-established triggers of senescence, even though senescence within the ependymal cell lining has not been specifically examined in multiple sclerosis.^134^ The relationship between senescence and ependymal cell dysfunction merits further research in order to better understand the precise mechanisms by which ependymal cell dysfunction occurs and the repercussions that it may incur in other brain regions.

## Conclusion

Ependymal cells form important cellular barriers that are critical in maintaining overall brain health. While disruption to the blood–brain barrier has been extensively studied in disease, compromised function of the CSF-brain and blood-CSF ependymal barriers have been critically overlooked, though they likely contribute to neurodegeneration as well. Damage or injury to ependymal cells have been shown to impair cilia function, dysregulate CSF dynamics and circulation, promote the secretion of pro-inflammatory factors, and potentially trigger pre-mature senescence. Due to the importance of CSF homeostasis in regulating normal brain activities, ependymal cell dysfunction has even been suggested as an early manifestation of certain neurodegenerative diseases. Consequently, this raises the question if ependymal cells may be a potential origin of disease and if dysfunction of the ependymal cell lining may lead to widespread dysfunction and neurodegeneration of the brain. Further research into this area may lead to the development of diagnostic methods and treatments that utilize biomarkers of ependymal cell dysfunction to detect and target earlier stages of specific neurodegenerative diseases.

## Data Availability

This review article did not involve the generation of new data.

## Abbreviations

ALS =: amyotrophic lateral sclerosis
IL =: interleukin
mTBI =: mild traumatic brain injury
NSCs =: neural stem cells
*PACRG* =: parkin co-regulated gene
PCM1 =: pericentriolar material 1
SASP =: senescence-associated secretory phenotype
SCO =: subcommissural organ
TBI =: traumatic brain injury
V-SVZ =: ventricular-subventricular zone
